# Management of Long-Term Sores and Ulcers of Breast Cancer Survivors With Chinese Herbal Medicines: A Case Report

**DOI:** 10.3389/fpsyt.2022.874691

**Published:** 2022-04-08

**Authors:** Yuanfeng Zhang, Bing Zhang, Weilong Zhou, Tao Lu

**Affiliations:** ^1^School of Chinese Materia Medica, Beijing University of Chinese Medicine, Beijing, China; ^2^China Traditional Chinese Medicine Science and Technology Development Center (Talent Exchance Center of National Administration of Traditional Chinese Medicine), Beijing, China; ^3^School of Life Sciences, Beijing University of Chinese Medicine, Beijing, China

**Keywords:** Chinese herbal medicine, sore and ulcer, cancer metastatic recurrence, psychosocial support, post-radiofrequency ablation wound, breast cancer survivors

## Abstract

**Background:**

The treatment of long-term sores and ulcers of breast cancer metastatic recurrence is a serious challenge with successful cases rarely being documented. Herein we reported a successful case using the internal vitality supporting method of Chinese herbal medicine (CHM).

**Case Summary:**

A 59-year-old female Chinese patient, 10 years after breast cancer surgery, developed metastatic lesions in the lung. Thereafter she received radiofrequency ablation and adjuvant treatments for 1 year with severe sequelae, a right unhealed sore and ulcer. She became frustrated and depressive. And subsequently sought exclusive treatment under the guidance of a Traditional Chinese Medicine (TCM) physician. The patient's condition was categorized as a *Qi* (or vitality) deficiency-related sore and ulcer. In the next six months, the patient still follows a traditional Chinese medicine therapeutic regimen based on the internal vitality supporting method of Chinese herbs.

**Conclusion:**

The sore and ulcer from the surgical wound were healed. Up to now, the tumor markers have remained stable. TCM personalized survivorship treatment and psychosocial support can help patients improve their quality of life after acute treatment and in the long-term for cancer survivors.

## Introduction

Breast cancer is the most common cancer diagnosed in women worldwide, and it alone accounts for 30% of cancer in women (1). Death rates for female breast cancers were estimated to be 6.9% in 2020 (2).

Surgery represents the only potentially curative therapeutic intervention for breast cancer patients, and postmastectomy locoregional recurrences (LRRs) were found in 5–25% (3). The sites of LRRs were usually confined to the chest wall (68%) and supraclavicular nodes (41%) (3, 4). The lung, bone, and liver were the most common metastatic sites for breast cancer. A total of 60–70% of metastatic breast cancer patients who eventually died were diagnosed with lung metastasis (5). Unique signal transduction pathways were found for breast tumors to metastasize to the lung, and nine genes have been defined as specific for lung-specific metastasis (6–8). Surgery is the first line choice of treatment. Other treatments (e.g., cryoablation, intraoperative radiotherapy, high-intensity focused therapy, and ultrasound) are used in the experimental/investigational phase, if applicable (3). Radiofrequency ablation (RFA) is a popularity intervention for its advantages of reduction in tumor mass and intraprocedural pain (9). However, adverse events are common with RFA, such as vascular injury (10), atrio-esophageal fistula (11), and deep esophageal ulcers and abscesses (12–15), which seriously affect patients' quality of life. At present, post-RFA wound management is a serious challenge, as the wound is difficult to heal and has to be kept clean by rising with saline water, which is the main conservative approach for wound management. According to traditional Chinese medicine (TCM) theory, *Yang* and *Qi* are the driving forces of biological activities in the human body. Deficiencies in *Yang* and *Qi* are common in cancer patients (16). Chinese herbal medicine (CHM) has been utilized as a supplementary treatment (17, 18) for breast cancer patients who received a surgical operation (19–21). We report herein the case of a 59-year-old women who had a surgical operation in the year of 2007. The patient was diagnosed with lung metastasis by CT, for which RFA treatment was then performed. Unfortunately, the cancer recurred and the chest ulcer failed to be controlled following interventional therapy. In the absence of further treatment options, the surgeon decided to offer only palliative treatment. The patient developed anxiety and depressed mood. Without further improvement, the patient turned to CHM for help, and she witnessed a stable improvement in her condition, with the sore and ulcer healed.

### Ethical Approval

This case report was a retrospective study; hence it did not belong to the Ethical committee's scope of review, which means that ethical approval was not necessary.

### Patient Consent

Written informed consent was obtained from the patient before and after every procedure.

## Case Presentation

### Chief Complaints

The patient was a 59-year-old woman diagnosed with right breast cancer who underwent a right mastectomy in 2007. The patient complained of persistent chest pain and ulcers for 1 year, and she had low quality of life.

### History of Present Illness

The patient was sent to Guoyitang TCM Clinic, Beijing University of Chinese medicine on 29 June 2017. She reported having developed an ulcer on the right side of her chest 1 year prior, after receiving RFA treatment in Guangdong hospital. The patient reported coughing with a lot of white sputum. She reported that she had persistent pain on the right side of her chest and there was secretion at the scar, and she had poor mental status. We observed that her tongue was pale-white-with tenderness and the tongue coating was white with exfoliative tongue fur. The patient's pulse was deep.

### History of Past Illness and Family History

In October 2007, the patient had an invasive ductal carcinoma that was diagnosed by puncture. Grade II was confirmed in the right breast tissue and lymph node via microscopy, with metastases involving the axillary lymph node (7) 4/7 and lymph node (9) 5/9 depicted in the surgical pathology report. Immunohistochemical analyses revealed the following: ER (+++), PR (+++), C-erbB-2 (-), VEGF (++), P53 (+++), and Ki-67 (+++). Having received postoperative radiotherapy, the patient continued taking toremifene as a means of endocrine therapy for 5 years.

In May 2016, a CT scan demonstrated a recurrence of right breast cancer and aroused suspicions of lung metastasis, after which ultrasound and CT-guided right recurrent breast lesions and radiofrequency ablation of the lung metastases were conducted. Following radiofrequency ablation of the lesions within the lung and right chest wall, the wound did not heal completely, and a chest wall sinus was formed. Next, the patient started taking tegafur gimeracil oteracil potassium capsules (20 mg per day) until January 2020. And is currently being administered fulvestrant injections.

In March 2017, the patient received a secondary radiofrequency ablation for the metastasized lesion in her left lung. Unfortunately, the patient complained of persistent chest pain and ulcers; a chest CT revealed a mass measuring 12 mm at its thickest point, suggesting the presence of recurrence and that the lesion was inoperable. The wound was covered with dressing, and the surgeon recommended the patient use a saline cleaner for the wound 1~2 times daily. However, in the patient's perspective, she said there has been almost no improvement. She felt that the condition gradually worsened. This clearly devastated the patient and seriously affected her quality of life.

**Figure 1:**
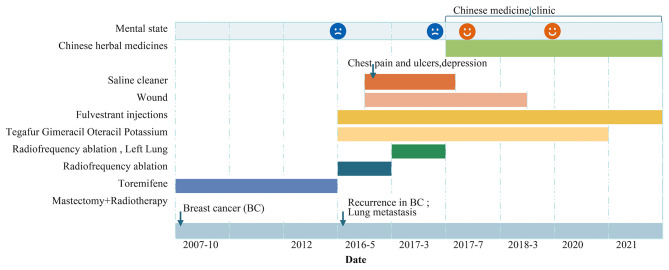
The timeline for medical history and treatment.

Regarding her psychosomatic symptoms, the patient had suffered chronically from dysthymia after cancer diagnosis. Depressive symptoms increased with cancer diagnosis and multiple RFA therapy regimes. And her psychiatric profile became worse when the wound was still unhealed after 1 year. The timeline for medical history and treatment is shown in Figure 1.

### Diagnostic Assessment and Treatment

**Figure 2:**
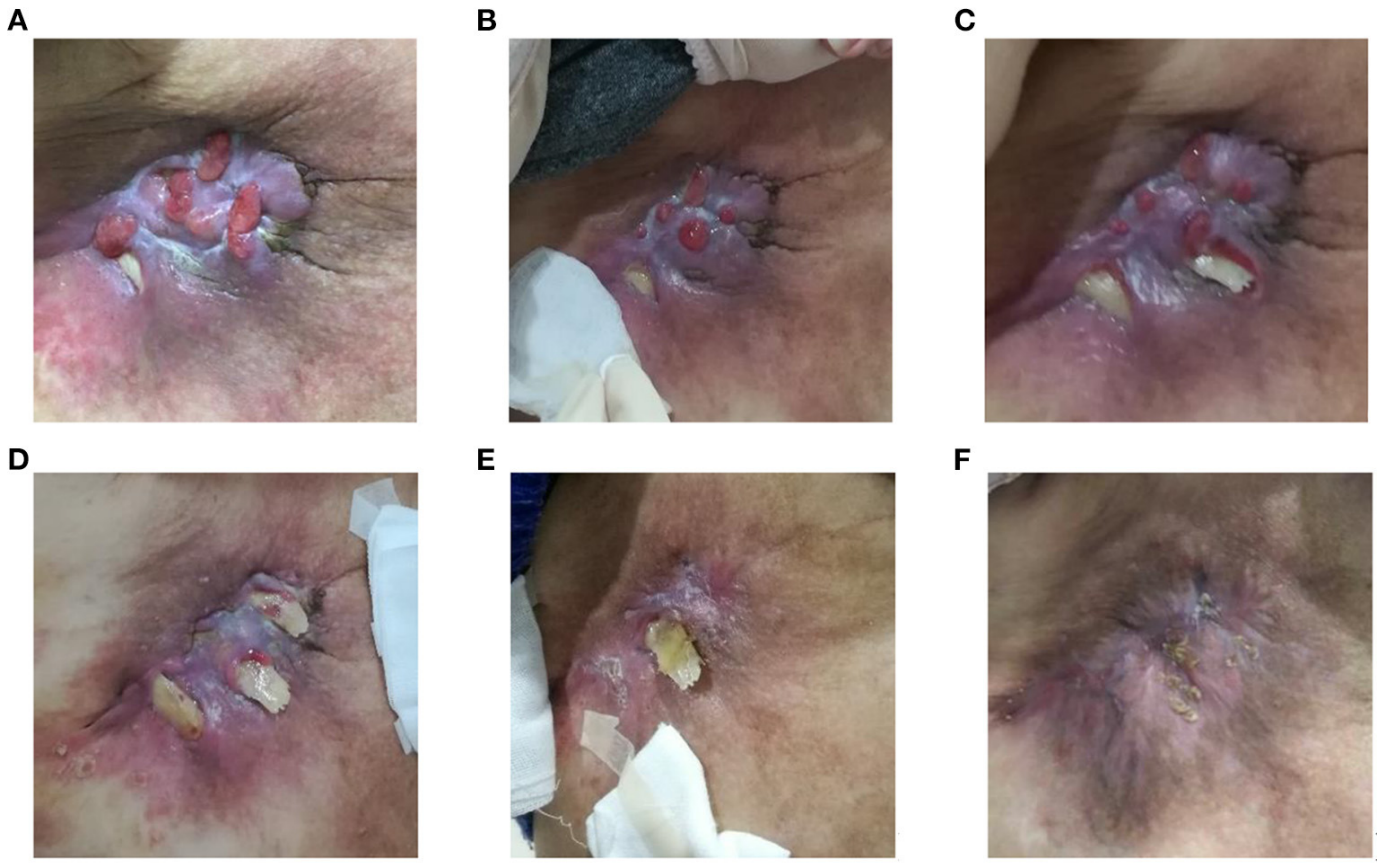
Sore and ulcers. **(A)** Severe sore at the first visit (with eight ulcers on right chest). **(B–E)** By the second and fifth visits, the sore and ulcers were significantly improved. **(F)** At the sixth visit, the ulcers had already healed.

According to TCM syndrome differentiation, the patient was diagnosed with *Yang (spleen and kidney)* deficiency and *Qi* deficiency. Professor Dr. Zhang's (Doctor of CHM) main treatment goal was to relieve the patient's pain. She used the internal vitality supporting method (22) to strengthen the patient's anti-pathogenic ability. A treatment course lasted for 7 days. The composition formula is displayed in the Supplementary Table. First, the patient was instructed to take the herbal decoction once a day for 7 days. At the second visit, the patient appeared cheerful and lively. The improvement elicited during the treatment was highly significant (Figure 2).

Currently, the patient continues to take Chinese herbs after completing five treatment courses. Over the last 6 months, the wound has healed and the overall situation has remained stable with a slight cough. “I was very surprised at my change because I could hang out easily at home with my family with a comfortable mind”, the patient said.

### Outcome and Follow-Up

In March 2018, the CT report pointed out that the lesion in the right chest wall with its thickest part measuring 9 mm, was smaller in size than previously reported (12 mm) in June 2017. The tumor marker test was sustained within the normal range (Table 1).

**Table 1:** Tumor marker test.

**Test item**	**Prior treatment**	**Post-treatment**	**Reference**	**Units**
	**with CHM**	**with CHM**		
CA15-3	14.17	13.11	0.00~25.00	U/ml
CA125	7.12	6.97	0.00~35.00	U/ml
CEA	1.42	2.02	0.00~5.00	ng/ml
CA19-9	–	21.43	0.00~35.00	U/ml

*CA15-3, carbohydrate antigen 153; CA125, carbohydrate antigen 153; CEA, carcinoembryonic antigen; CA19-9, carbohydrate antigen 199*.

## Discussion

There are three stages in the wound healing process: inflammation, proliferation, and remodeling, which depend on the host's ability to repair tissue (23). It was proposed that there was an aspect of surgical tumor resection that triggered the outgrowth of otherwise-dormant metastases, leading to the synchronous pattern of relapse during the postoperative period. In general, 10~41% of breast cancer patients experience recurrences, and 10% develop overt metastases relatively soon after tumor eradication (3, 24). There is compelling evidence of an increased risk of anxiety, depression, and suicide, and neurocognitive and sexual dysfunctions in breast cancer survivors compared with women with no prior cancer (25). Clinically relevant symptoms of stress and depression are common shortly after diagnosis and metastatic recurrence, because women feel uncertainty about the future and their cancer may be perceived as life-threatening. The patient mentioned in this case report who suffered from long-term sores and ulcers of breast cancer metastatic recurrence had low quality of life. From the surgeons' perspective, ulcers of breast cancer metastatic recurrence are local inflammation, and they only recommend washing the ulcer with normal saline, but in this case, the ulcer failed to heal for 1 year. This case was complex and difficult to treat. Seeking the help of a doctor was spiritually necessary for the patient. So when the surgeon was helpless or the treatment plan was not ideal, this led to despair in the patient.

The holistic concept is an essential philosophy of TCM. The mind and body together are a dynamic battlefield, in which all the organs and mind are interconnected and bound to each other, and should be considered and treated as a whole, rather than an isolated pathology. In TCM theory, individual health is formed by *Yin-Yang*; an imbalance between *Yin* and *Yang* in the body can render an individual more prone to certain disease (26). This theoretical advantage simplifies the complexity. TCM uses different methodologies to diagnose mental and body disorders. The four diagnostic methods (inspection, listening/smelling, inquiry, and palpation) are the means to obtain patient information. This information is then analyzed using diagnostic models, such as yin/yang, interior/exterior, excess/deficiency, and hot/cold to differentiate the pattern and form a diagnosis. Zhang suggested that the patient's long-term sore and ulcer were due to *Qi* deficiency; the psychiatric symptoms of the long-term sore and ulcer resulted in emotional upset and stagnation of *Qi*. These were associated with the impairment of spleen and kidney (*Spleen-Yang* and *Kidney-Yang*). *Yang*-deficiency (e.g., *spleen, kidney*) may not only be related to hypothalamic-pituitary-adrenal (HPA) axis and hypothalamic-pituitary-thyroid (HPT) axis dysfunction, but also to functional disorders of the cyclic nucleotide and immune systems (27). These factors are involved in depression. The patient's mind and body were affected by the sore and ulcer. Zhang's main treatment goal was to relieve the patient's pain, which could improve the patient's quality of life.

After the malignant lesions had been removed by surgical excision, there was a persistent poor growth of granulation tissue, following delayed wound healing. The patient exhibited middle-staged boils and sores, and the pus was not completely discharged after breaking the ulceration. The patient had a compromised immune function; thus a tonic was used for reinforcing the primordial *Qi* in order to treat suppurative infection in this situation. This method was named the internal vitality supporting method according to the theory of TCM (22). This therapeutic regimen aimed at strengthening the host's anti-pathogenic ability and removing pathogenic factors via herbs to achieve the goal of canceling the tumefaction and shrinking its base to avoid ulceration.

The ulcer was effectively healed by Chinese herbs via boosting the primordial *Qi*, conditioning the internal vitality of key viscera to circulate *Qi*, activating the energy supply, and dispersing the inflammatory blockage. HuangQi (*Radix astragali seu Hedysari*), DangShen (*Radix codonopsis pilosulae*), Baizhu (*Rhizoma Atractylodis Macrocephalae*), Danggui (*Radix Angelicae Sinensis*), Baizhi (*Radix Angelicae Dahuricae*), etc., have been documented to modulate the immune response. These were used in inhibiting cancer cell proliferation, arresting the cell cycle, inducing apoptosis, inhibiting epithelial-mesenchymal transition, regulating immune function (28), and exerting antioxidative and anti-inflammatory effects (29) in cells and animal studies. Compared to local treatment therapies for chronic inflammatory skin disease, this herb intervention altered the gut microbiome, taking advantage of the gut-skin axis to control different skin conditions (30). As these herbs encompass a group of substances that produce a therapeutic effect, multiple pathways and receptors were targeted, plenty of compounds were involved, which acted synergistically to improve cancer-related symptoms, enhance vital energy, and boost immunity. What is more, herbs used in traditional medicine can be significantly effective in reducing depression, depressive symptoms, and improving patients' performance via regulating the HPA axis (31). So as the patient witnessed her wound healing, she became more optimistic.

This case was the first description of management of a long-term sore and ulcer of breast cancer metastatic recurrence with the internal vitality supporting method of Chinese herbal medicine. The patient was effectively cured due to cross-disorder and interdisciplinary collaboration. The holistic concept of TCM provided a novel therapeutic strategy for mind–body medicine.

## Data Availability Statement

The original contributions presented in the study are included in the article/Supplementary Material, further inquiries can be directed to the corresponding author/s.

## Ethics Statement

Written informed consent was obtained from the relevant individual(s) for the publication of any potentially identifiable images or data included in this article. Written consent and accompanying photos were obtained from the patient and her husband for publication.

## Author Contributions

YZ drafted the manuscript. BZ treated the patient and provided the case analysis. WZ and YZ provided the critical analysis in preparing this article. TL revised the manuscript. All the authors approved the final version.

## Funding

This work was supported by the Programs Foundation for Leading Talents in State Administration of Traditional Chinese Medicine of China-Qihuang Scholars Project (10400633210004), the National Natural Science Foundation of China (Grant No. 81874349), and National Special Support Plan for High-Level Talents (Plan of ten thousand people)-Famous Teacher Program to BZ.

## Conflict of Interest

The authors declare that the research was conducted in the absence of any commercial or financial relationships that could be construed as a potential conflict of interest.

## Publisher's Note

All claims expressed in this article are solely those of the authors and do not necessarily represent those of their affiliated organizations, or those of the publisher, the editors and the reviewers. Any product that may be evaluated in this article, or claim that may be made by its manufacturer, is not guaranteed or endorsed by the publisher.

## Abbreviations

TCM: Traditional Chinese medicine
LRRs: Locoregional recurrences
RFA: Radiofrequency ablation
CT: Computed tomography
CHM: Chinese herbal medicine.
